# Utilizing Structural Equation Modeling–Artificial Neural Network Hybrid Approach in Determining Factors Affecting Perceived Usability of Mobile Mental Health Application in the Philippines

**DOI:** 10.3390/ijerph19116732

**Published:** 2022-05-31

**Authors:** Nattakit Yuduang, Ardvin Kester S. Ong, Nicole B. Vista, Yogi Tri Prasetyo, Reny Nadlifatin, Satria Fadil Persada, Ma. Janice J. Gumasing, Josephine D. German, Kirstien Paola E. Robas, Thanatorn Chuenyindee, Thapanat Buaphiban

**Affiliations:** 1 School of Industrial Engineering and Engineering Management, Mapúa University, 658 Muralla St., Intramuros, Manila 1002, Philippines; nuttakit33@gmail.com (N.Y.); aksong@mapua.edu.ph (A.K.S.O.); nbvista@mymail.mapua.edu.ph (N.B.V.); mjjgumasing@mapua.edu.ph (M.J.J.G.); jdgerman@mapua.edu.ph (J.D.G.); kperobas@mymail.mapua.edu.ph (K.P.E.R.); thanatorn_chu@rtaf.mi.th (T.C.); 2 School of Graduate Studies, Mapúa University, 658 Muralla St., Intramuros, Manila 1002, Philippines; 3 Department of Industrial Engineering and Management, Yuan Ze University, 135 Yuan-Tung Road, Taoyuan 32003, Taiwan; 4 Department of Information Systems, Institut Teknologi Sepuluh Nopember, Kampus ITS Sukolilo, Surabaya 60111, Indonesia; reny@its.ac.id; 5 Entrepreneurship Department, BINUS Business School Undergraduate Program, Bina Nusantara University, Jakarta 11480, Indonesia; satria.fadil@binus.ac.id; 6 Department of Industrial Engineering and Aviation Management, Navaminda Kasatriyadhiraj Royal Air Force Academy, Bangkok 10220, Thailand; thapanat_bua@rtaf.mi.th

**Keywords:** mobile mental health application, mental health, technology acceptance model, artificial neural network

## Abstract

Mental health problems have emerged as one of the biggest problems in the world and one of the countries that has been seen to be highly impacted is the Philippines. Despite the increasing number of mentally ill Filipinos, it is one of the most neglected problems in the country. The purpose of this study was to determine the factors affecting the perceived usability of mobile mental health applications. A total of 251 respondents voluntarily participated in the online survey we conducted. A structural equation modeling and artificial neural network hybrid was applied to determine the perceived usability (PRU) such as the social influence (SI), service awareness (SA), technology self-efficacy (SE), perceived usefulness (PU), perceived ease of use (PEOU), convenience (CO), voluntariness (VO), user resistance (UR), intention to use (IU), and actual use (AU). Results indicate that VO had the highest score of importance, followed by CO, PEOU, SA, SE, SI, IU, PU, and ASU. Having the mobile application available and accessible made the users perceive it as highly beneficial and advantageous. This would lead to the continuous usage and patronage of the application. This result highlights the insignificance of UR. This study was the first study that considered the evaluation of mobile mental health applications. This study can be beneficial to people who have mental health disorders and symptoms, even to health government agencies. Finally, the results of this study could be applied and extended among other health-related mobile applications worldwide.

## 1. Introduction

Mental health is a state of well-being wherein an individual realizes their own abilities, can cope with the normal stresses of life, can work productively, and is able to contribute to its community. According to the World Health Organization (WHO), mental health is an integral and essential component of health. It is fundamental to one’s collective and individual ability as a human to think, emote, interact with others, earn a living, and enjoy life. Thus, the promotion, protection, and restoration of mental health must be regarded as a vital concern of individuals, communities, and societies throughout the world [1].

In recent years, mental health has emerged as one of the most important public health concerns [2,3]. Mental illnesses such as depression, anxiety disorder, and mood disorder among others have been prevalent in countries worldwide. Among all the mental disorders, depression and anxiety disorder are the most common problem. Globally, an estimated 264 million people are affected by depression—among which 20% are children and adolescents [2]. Moreover, it is estimated that 264 million adults around the globe have anxiety disorders [4]. Overall, it was found that the prevalence of all mental disorders has increased by 50% worldwide from 416 million to 615 million between 1990 and 2013 [5]. One of the countries where mental illness is prevalent is the Philippines.

In the Philippines, mental illness is the third most common disability [3]. With an estimated 6 million Filipinos who are living with depression and/or anxiety, the Philippines was tagged as the third country with the highest rate of mental health problems across the Western Pacific Region [4]. It was explained from the Study by Martinez et al. [3] that approximately 3–4 in 100,000 people die due to suicide from mental health problems. However, it was deemed to be higher due to unreported cases. Moreover, the WHO Special Initiative for Mental Health in the Philippines which conducted research in the early part of 2020 showed that at least 3.6 million Filipinos suffer from one kind of mental, neurological, and substance abuse disorder [6].

At the present time, the COVID-19 pandemic has further impacted the mental health of young and older adults, health care providers, and people with underlying health conditions. During the COVID-19 pandemic, mental health and substance use issues have increased. The pandemic induced general stress which led to new mental health and substance use issues. As the pandemic continues, populations including that of the Philippines are at increased risk of experiencing poor mental health [7]. Accordingly, data show that the number of Filipinos affected by mental illnesses has tremendously increased. An assessment of the Philippines’ mental health system reported a 16% prevalence of mental disorders among children [2,8]. In addition, the latest Global School-Based Student Health Survey found that 16.8% of students aged 13–17 had attempted suicide one or more times during the 12 months before the survey [2].

Despite the increasing number of mental health problems in the country, it is one of the problems that is given less attention. In fact, the government only spends a total of 0.22% of its expenditure on mental health, accompanied by a lack of health professionals working in the mental health sector [9]. To date, it was estimated that the National Center for Mental Health accounts for 67% of the available psychiatric beds nationally [10]. Moreover, recent data have shown that there are only 1.08 mental health beds in general hospitals and 4.95 beds in psychiatric hospitals per 100,000 of the population [9]. In line with this, there are 46 outpatients’ facilities and four community residential facilities. As for the psychiatric hospitals, there are only two existing hospitals in the country—the National Center for Mental Health situated in Mandaluyong and Mariveles Mental Hospital situated in Bataan. These two tertiary care psychiatric hospitals have 4200 beds and 500 beds, respectively [9].

There is also a scarcity of mental health staff in the country. While the WHO recommends a global target of 10 psychiatrists per 100,000 population, the Philippines only has one doctor available for every 80,000 Filipinos [9]. Moreover, a little over 500 psychiatrists are in practice in the country. The ratio of mental health workers per population in the Philippines is low at 2–3 per 100,000 population [11]. Thus, these figures only prove that there is a severe shortage of mental health facilities, health care programs, and staff in the country.

Apart from the problem of the shortage of mental health services in the country, another problem that the country is facing is the barrier of help-seeking behavior in Filipinos due to internalized stigma of mental health disorders [12]. Most Filipinos experience difficulty in admitting to themselves that they are in need of help when it comes to their mental health status. There is evidence indicating that most Filipinos are reluctant in seeking professional help for their mental health issues. In the survey conducted by Tuliao and Velasquez [12], among 359 Filipino college students in the Philippines, only 22% had sought professional help for an academic or non-academic issue in their lifetime. In addition, there was a significantly higher preference to seek help from friends and family members than from professional counselors and psychotherapists.

Apart from the stigma surrounding mental health, another barrier to seeking professional help is the cost of therapy and medication. In Metro Manila, the cost of therapy per session ranges from PHP 1000 to PHP 4500 (USD 19.10–USD 85.95) which is deemed expensive due to the minimum income of the Filipinos. Depending on the case, the frequency of a patient visiting hospital and how much they spend on expensive medication varies. Known brands such as Xanax and Prozac cost approximately PHP 130 per tablet (USD 2.48) while other anti-depressants and anti-psychotic drugs can cost as much as PHP 300 each (USD 5.73). Since maintenance is needed among the medication, this caused hesitance among Filipinos to consider treatment.

Due to the increasing number of mental health issues, mobile mental health applications have emerged in the present time. There has been increasing interest in the use of smartphone applications and other consumer technology in mental health care for a number of years [13]. There are now different mobile mental health applications such as Headspace, MindMission, Happify, and Lusog-Isip App, among others, that aim to provide assistance to mild to normal symptoms of mental health problems such as depression, anxiety, and mood disorder, among others. These mental applications help users better understand their current feelings and emotions, ease the symptoms that they are experiencing, deal with stress, and practice appropriate exercises for a certain situation. This is in line with the RenewHealth program being circulated in the Philippines [14]. Thus, the existence of such mobile mental health applications can be one of the solutions to meet the demand for mental health treatment. Given that most Filipinos now use smartphones, accessibility to such treatment will now be within reach. In addition, with this, problems regarding cost and time will be at ease.

This study aimed to analyze the acceptance and intention of the Filipino population to use mobile mental health treatment, measured through the use of the extended technology acceptance model (TAM). Different factors such as social influence, service awareness, technology self-efficacy, perceived usefulness, perceived ease of use, convenience, voluntariness, user resistance, intention to use, actual use, and perceived usability were considered in this study. The integration of a structural equation modeling (SEM) and artificial neural network (ANN) hybrid was utilized to evaluate the findings of this study. The results in this study seek to determine the different factors affecting the intention to use and perceived usability. With this, it would help promote the usage and patronage of the mental health application available. The result of this study will help academics, stakeholders, and developers to enhance the mobile application for continuous utility to help people with mental health problems. Moreover, this study could be applied and extended to assessing mobile health applications worldwide.

## 2. Literature Review and Conceptual Framework

### 2.1. Technology Acceptance Model

**Figure 1:**
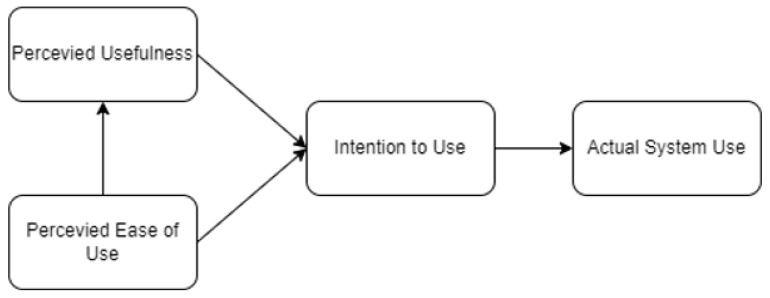
Technology acceptance model [20].

The technology acceptance model (TAM), presented in Figure 1, is a framework used to measure popular acceptance of newly available technologies [7]. Kamal et al. [7] added that it is crucial to evaluate human factors in assessing their willingness to accept and utilize a certain technology for it to be successful. Studies relating to mobile health adaptation in different countries have considered TAM and its extension. Tubaishat et al. [15] considered extending TAM in measuring usefulness and ease of use among e-health records for nurses in Jordan. Their study provided insights into how both the perceived usefulness and perceived ease of use affected acceptance and continuous intention to utilize the mobile application. Tsai et al. [16] considered an extended TAM for evaluating factors affecting the acceptance and resistance of telehealth in Taiwan. Their results explained how the availability of the application and its usefulness were key factors for predicting acceptance; otherwise, resistance would be evident. However, these studies solely utilized Multiple Linear Regression and SEM alone.

On the other hand, different theories were evaluated by Sohn and Kwon [17] utilizing artificial intelligence. Among the theories, TAM, theory of planned behavior, value-based adoption model, and the unified theory of the acceptance and use of technology was evaluated. Behavioral-related studies would lead more to VAM for a proper theoretical framework [17]. However, Talukder et al. [18] presented how integrating SEM and ANN would be beneficial in assessing health-related technology acceptance. It could therefore be highlighted that TAM may be utilized for assessing the characteristics involving human factors [19], especially with the integration of SEM and ANN.

### 2.2. Conceptual Framework

**Figure 2:**
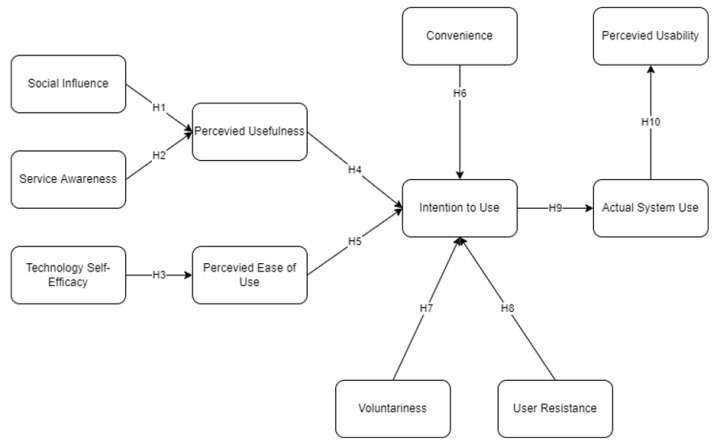
Conceptual research framework.

Figure 2 represents the conceptual framework of this study. The study considered an extended technology acceptance model to determine the factors affecting the perceived usability of a mobile mental health application in the Philippines during the COVID-19 pandemic. Factors such as social influence, service awareness, technology self-efficacy, perceived usefulness, perceived ease of use, convenience, voluntariness, user resistance, intention to use, actual use, and perceived usability were assessed, wherein 10 hypotheses were created to be evaluated utilizing an SEM–ANN hybrid.

Social influence is defined as the effect that other people have on an individual’s decisions, in this case in relation to technology use. The study by Min et al. [21] showed how social influence is a significant factor when it comes to utilizing mobile applications for transportation. In the case of technology, compatibility plays an important role in a person’s perception [22]. Campbell and Russo [22] added how the interaction of an individual through social context causes a significant impact, either positive or negative, on a person’s intention to use a mobile application. Moreover, Walrave et al. [23] showed how social influence is a significant factor when it comes to the intention to use a mobile health-related application. Thus, it was hypothesized that:

**Hypothesis** **1** **(H1).** *Social influence has a direct positive significant effect on perceived usefulness.*

Service awareness in this study implies the recognition of the available application or platform. Safi et al. [24] presented how the acceptance of how useful an application is dependent on the users’ awareness. It was added that if the users are aware and have the autonomy to decide, then the positive acceptance of the application is more likely. In relation to this is technology self-efficacy. Technology self-efficacy is the ability of an individual to accomplish tasks using an application such as e-health technologies [25]. Their technical skills and knowledge should be implied to have a positive effect towards their perception of usefulness and perceived ease of use. In addition, Talukder et al. [18] explained how technological fears and low interest in using the application would lead to a negative effect on people’s person of usefulness and ease of use. Moreover, Meuter et al. [26] presented how elderly people that are not technology inclined have a negative perception of mobile application usage. Therefore, the following was hypothesized:

**Hypothesis** **2** **(H2).** *Service awareness has a direct positive significant effect on perceived usefulness.*

**Hypothesis** **3** **(H3).** *Technology self-efficacy has a direct positive significant effect on perceived ease of use.*

Under TAM, factors such as perceived usefulness and perceived ease of use are primary latent variables. Tsai et al. [16] presented how the availability of resources and perceived usefulness have a great positive impact on the intention to use a health-related mobile application. It was implied that when people find it easy to use, then less technology anxiety is observed, and thus a higher acceptance to patronize the mobile application will be evident. In addition, Tubaishat [15] presented how both the perceived usefulness and perceived ease of use would lead to a positive significant effect on the intention to use when people have the necessary technical skills. Lee et al. [27] also presented that aside from social interaction, the usefulness of a health-related application would have a positively significant effect on intention to use. Therefore, it was hypothesized that:

**Hypothesis** **4** **(H4).** *Perceived usefulness has a direct positive significant effect on intention to use.*

**Hypothesis** **5** **(H5).** *Perceived ease of use has a direct positive significant effect on intention to use.*

Convenience in this study considered how beneficial the mobile application is—even under undesirable conditions. Under TAM, convenience is an extension variable among studies that have considered evaluating the intention to use and acceptance among users. Sun and Zhang [28] emphasized the positive effect of a mobile application has when it comes to continuous usage. In support, Shin [29] presented how hedonism has an effect on the intention to use a mobile application. Thus, it was hypothesized that:

**Hypothesis** **6** **(H6).** *Convenience has a direct positive significant effect on intention to use.*

In this study, voluntariness is the ability to decide either through one’s own or others’ influence to utilize a mobile application. Okumus et al. [30] presented how the availability of technology nowadays has become part of the daily utility of an individual. Lu et al. [31] explained how the positive mobility drive of an individual would lead to the positive intention of using technology. Tamilmani et al. [32] showed that when a mobile application has been deemed significantly helpful in daily activities, then people would have continuous intention to use it. Thus, it was hypothesized that:

**Hypothesis** **7** **(H7).** *Voluntariness has a direct positive significant effect on intention to use.*

User resistance has been considered when it comes to technology acceptance as a factor that may influence people’s intention to use a system or application. Tsai et al. [16] presented that transition cost and technology anxiety would lead to resistance to using technology. Sohn and Kwon [17] presented how characteristics from combined products in artificial intelligence would promote reduced resistance to use. Since this study considered a significantly new mobile application that is intended for mental health, then the development and utility that the application has are relatively new. Therefore, the inclusion of this factor is crucial with regard to the user’s intention to use the mobile application. Thus, it was hypothesized that:

**Hypothesis** **8** **(H8).** *User resistance has a direct positive significant effect on intention to use.*

Intention to use has been said to affect actual system use. Wu and Du [33] explained how the intention to use a technology is positively correlated with the actual system use. As the intention is the performance of a particular action, when technology is perceived as satisfactory among users due to its positive influence on the health of the user, then it would lead to the actual system usage [34]. Different studies have presented a positive influence on the intention and actual system use when it comes to health-related mobile applications. Dehghani [35] showed how a positive influence is seen when dealing with goods, health-related activities, and enabling technologies. Moreover, Huang and Yang [36] presented the positive relationship between the intention to use and actual system use when dealing with health-related technologies. Therefore, it was hypothesized that:

**Hypothesis** **9** **(H9).** *Intention to use has a direct positive significant effect on actual system use.*

Actual system use has been identified as a factor affecting perceived usability. There have been studies [37,38] that showed how perceived usability is positive when users have positive intentions, leading to actually utilizing a system. Wu and Du [33] showed a positive correlation between actual system use and perceived usability. Huang and Yang [36] also showed how perceived usability is preceded by actual system use due to the positive effect of people’s intention to use any health-related technology applications. Thus, it was hypothesized that:

**Hypothesis** **10** **(H10).** *Actual system use has a direct positive significant effect on perceived usability.*

## 3. Methodology

### 3.1. Participants

Due to the current COVID-19 pandemic, an online survey was utilized in this study. Table 1 shows the general characteristics of the participants. Among the 251 respondents of the conducted survey, 51% were female and 49% were male with ages ranging from 18 to 25 years old (87.25%). The demographic questions revealed that these were both students (52.99%) and workers (47.01%). Additionally, Estrada et al. [2] showed how younger generations are those which are really affected by mental health challenges. Moreover, not all individuals in the Philippines are open to talking about mental-health-related issues due to the stigma it has in the country [1]. The majority had finished a bachelor’s degree (68.92%) or secondary education (senior high school; 22.71%). Among the respondents, 96.02% had easy access to internet. However, 75.70% had no experience of online treatment while 24.30% had experienced online treatment. This shows how little knowledge people have about treatments available online.

### 3.2. Questionnaire

In the constructed questionnaire, each indicator was adapted from a study in the literature, as presented in Table 2. A total of 42 constructs to measure different factors were considered in this study. The questionnaire had items to measure latent variables such as social influence (SI), service awareness (SA), self-efficacy (SE), perceived usefulness (PU), perceived ease of use (PEOU), convenience (CO), voluntariness (VO), user resistance (UR), intention (IU) to use, actual system use (ASU), and perceived usability (PRU). Prior to the distribution of the questionnaire, a preliminary run was conducted and a Cronbach’s alpha value of 0.804 was obtained.

### 3.3. Structural Equation Modeling

To measure factors affecting technology acceptance, structural equation modeling (SEM) was utilized in this study in accordance with related literature using SPSS AMOS 26. Prasetyo et al. [42] utilized SEM to measure factors affecting the acceptance of online learning platforms. Their result showed a positive outcome with the integration of TAM and DeLone and the McLean IS Success Model. Moreover, Duarte and Pinho [43] utilized SEM to evaluate mobile adoption among people in Portugal. However, their study suggested considering the integration of other tools with SEM to highlight the results due to the SEM limitation. Supported by the discussion made by Fan et al. [44], SEM has limitations due to the indirect effects that one latent variable has on another. In this case, the theoretical framework extended the TAM which resulted in indirect effects presented in Figure 2. Thus, an artificial neural network was integrated to verify and highlight the results of this study.

### 3.4. Artificial Neural Network

ANN has been considered a powerful tool which has adopted how the human brain receives responses from neurons in the body. Different studies have considered ANN for evaluating and predicting human behavior worldwide [45,46]. ANN has been considered in this study by utilizing Python 5.1. Initial optimization to determine the best activation function for the hidden layer, output layer, number of nodes, optimizer, and training and testing ratios were conducted. A total of 10,542 datasets were considered in the feed-forward ANN optimization. Data cleaning utilizing correlation analysis was performed considering values with a correlation coefficient greater than 0.20 and a *p*-value of less than 0.05. Subsequently, we performed data aggregation and data normalization. Pradhan and Lee [47] considered 10 runs for each combination with 150 epochs [48] and thus this was adopted in this study. The optimum result presented an average accuracy of 86.12% for the model with 20 nodes, Tanh as the hidden layer activation function, Softmax for the output layer, and Adam as the optimizer for the 80:20 training and testing ratio.

## 4. Results

### 4.1. Structural Equation Modeling Results

**Figure 3:**
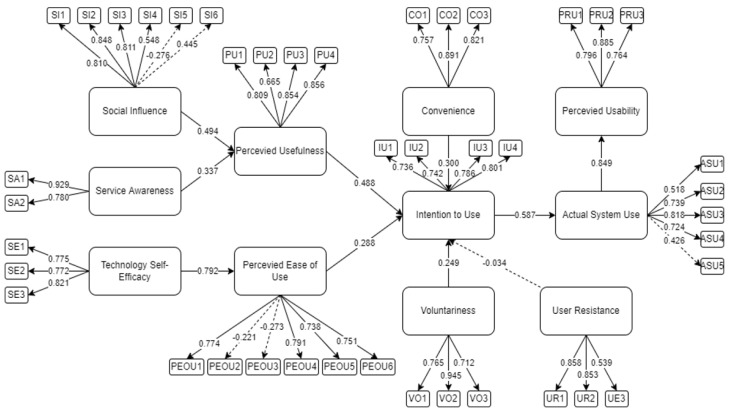
The final SEM to determine factors affecting the perceived usability of a mobile mental health application.

Figure 3 depicts the initial SEM to determine the factors affecting the perceived usability of mobile mental health applications among Filipinos during the COVID-19 pandemic. As seen in Figure 3, only user resistance (H8) did not meet the criteria of being significant (*p*-value > 0.05). In addition, SI5, SI6, PEOU2, PEOU3, and ASU5 indicators have values less than the 0.05 threshold [25]. Therefore, a revised model was constructed, omitting the insignificant variables to enhance the model fit of this study.

**Figure 4:**
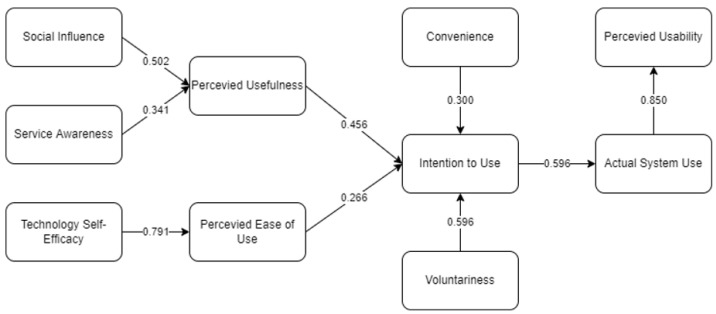
The final SEM to determine the factors affecting the perceived usability of a mobile mental health application.

Figure 4 presents the final SEM for factors affecting the perceived usability of mobile mental health applications during the COVID-19 pandemic. It could be deduced that 9 out of 10 hypotheses were considered significant (*p*-value < 0.05). Among these, resistance to use was removed which indicates that people are open and can utilize this mobile application for their mental health. Moreover, Table 3 presents the descriptive statistics of the indicators including the results of the initial and final factor loading.

The model fit of the result was also considered with parameters such as IFI, TLI, CFI, GFI, and AGFI. Presented in Table 4, a minimum cut-off of 0.80 was considered following the suggestion of Gefen et al. [49]. Moreover, the root mean square error with a value of less than 0.07 was observed [50,51]. This indicates that the model considered in this study is acceptable.

Finally, Table 5 presents the causal relationship of the considered framework. It could be deduced that ASU on PRU had the highest significant effect, followed by SE on PEOU, IU on ASU, VO on IU, SI on PU, PU on IU, SA on PU, CO on IU, and PEOU on IU. Further verification of the relationship was conducted utilizing ANN.

### 4.2. Artificial Neural Network Results

**Figure 5:**
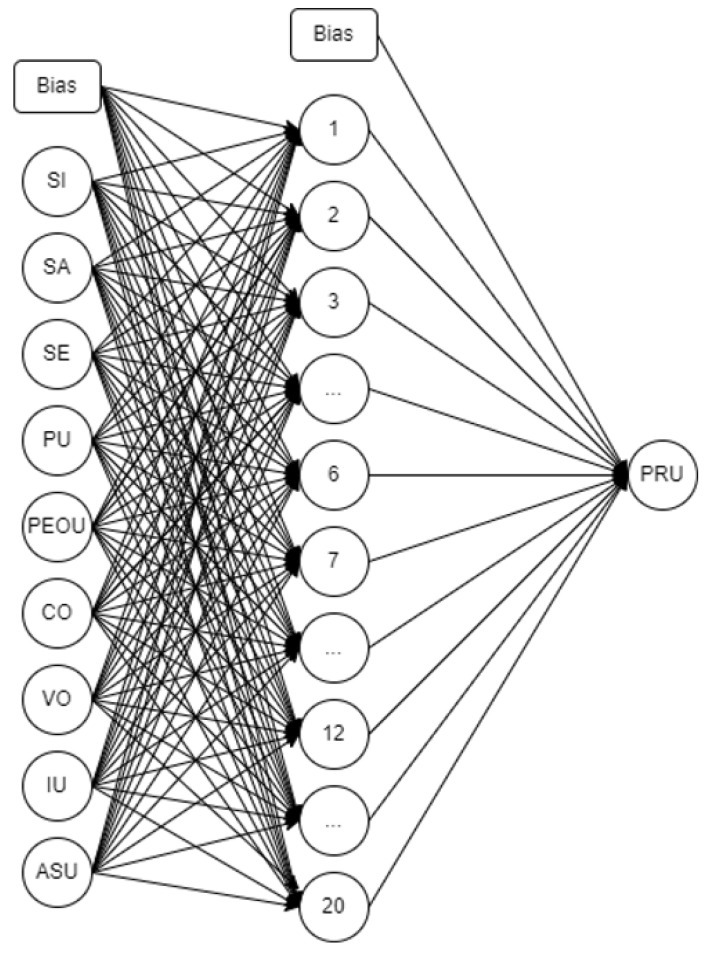
Artificial neural network model.

The ANN model considered in this study is presented in Figure 5. Similarly to the SEM result, RU was deemed insignificant (*p*-value > 0.05), which was removed. SI, SA, SE, PU, PEOU, CO, VO, IU, and ASU were considered the input nodes for the ANN. With the optimum parameters considered (Tanh, Softmax, Adam), the final result of the model showed an average prediction accuracy of 89.21%.

The results of ANN presented that VO showed the most important factor, followed by CO, PEOU, SA, SE, SI, IU, PU, and ASU. Although both results of SEM and ANN presented the same significant findings, it was deduced that the mediating effect and the indirect effect played a huge role in the results presented by the SEM [44,52]. Thus, a verification using ANN was conducted and the importance score rating among latent variables is presented in Table 6. Both presented that the user resistance was not significant. Therefore, the significant findings were verified by the SEM–ANN hybrid.

## 5. Discussion

The present study aimed to determine the factors affecting the perceived usability of mobile mental health applications. Using the SEM approach, latent variables such as social influence (SI), service awareness (SA), self-efficacy (SE), perceived usefulness (PU), perceived ease of use (PEOU), convenience (CO), voluntariness (VO), user resistance (UR), intention to use (IU), actual system use (ASU), and perceived usability (PU) were examined. Utilizing hybrid SEM–ANN methodology, 9 out of 10 hypotheses were considered significant.

Based on the result, UR was seen to be insignificant. It was seen that people are open to the new technology in coping with mental health stress when it comes to check-ups and consultations. Moreover, it was seen that people are open to having an alternative to the traditional therapy session available. Tsai et al. [16] supported this result. In their study, they showed how the availability of e-health services would lead to the intention to use an application. Tsai et al. [16] added that when people find it useful and advantageous (health-related), users would lead to the positive adaptation of e-health services such as mental health diagnoses. In addition, Safi et al. [24] showed how healthcare professionals are generally welcoming of new technology and presented how they find it advantageous as an additional means of easily interacting and caring for their patients.

This led to the highest importance score (100%) and significant latent variable of VO (β: 0.596; *p* = 0.011). It was shown that people are willing to use the application (at their own will) when it is introduced to them. Awareness of the application, knowing its benefits, and easy availability lead to a positive intention of using the application [53]. Lallmahomed et al. [54] also presented how easy access to available technology and access to Internet connection would lead to a positive influence of people’s intention to use it. This result is also supported by Alalwan et al. [55], who found that its usage depends on people’s perception of its benefit and utility. Thus, there is a positive effect of VO on mobile application use. This justifies the indirect effect of VO on ASU (β: 0.152; *p* = 0.012) and PRU (β: 0.129; *p* = 0.010).

The second-highest score of importance was seen with CO (78.1%), which has been seen as a significant factor affecting IU (β: 0.300; *p* = 0.009). Users find the mobile application convenient as they can use it anytime and anywhere, and it was seen to be highly useful even under undesirable conditions. Wu et al. [56] presented how a positive effect is seen on the IU of people when utilizing an application that they find useful and advantageous in their daily lives, especially when the application is health related. This is also supported by Nikolopoulou et al. [57], who found that the usefulness of technology would lead to continuous intention to use the technology, which will lead to actual usage. Thus, a positive indirect effect is seen with CO on ASU (β: 0.179; *p* = 0.009) and PRU (β: 0.152; *p* = 0.008).

Third, PEOU (65.3%) was seen to be a significant factor affecting IU (β: 0.266; *p* = 0.036). Based on the indicators, people find it beneficial, easy to utilize (technology wise), user-friendly and fool-proof. This result is verified by the study by Tsai et al. [16], who presented how the availability of an application relating to health is preceded by PEOU, which will positively affect IU. Moreover, Venkatesh et al. [58] explained how a system’s ease of use which affects a person’s intention to use will lead to them actually using the system. Thus, a positive indirect effect is seen for PEOU on ASU (β: 0.158; *p* = 0.036) and PRU (β: 0.135; *p* = 0.032).

Fourth, SA (58.4%) was seen to be a significant factor affecting IU (β: 0.341; *p* = 0.003). People are aware of its existence and they are also informed of the different applications available in relation to mental health applications. Palau-Saumell et al. [59] presented how the availability of resources would lead to an individual’s recognition in developing their habit of using it. Wu et al. [56] verified the findings of this study in which continuous usage due to awareness develops the habit of using the health-related application. In this case, there is an increase in IU when people are aware of its availability. This would lead to an indirect effect on ASU (β: 0.093; *p* = 0.005) and PRU (β: 0.079; *p* = 0.002).

In addition, SE was seen to have the fifth-highest score of importance (56.0%), as well as a significant direct effect on IU (β: 0.791; *p* = 0.012). Users are able to find the information and advice needed in mobile health applications, have the necessary skills to use the application, and be confident in regularly using it. This has led to an indirect effect on ASU (β: 0.125; *p* = 0.030) and PRU (β: 0.106; *p* = 0.028). When users are aware of the health risks, there would be a high intention of using the application [60]. In relation, Thakur [61] explained the relation of SE to the system or technology adaptation. This happens when people have already adopted the use of the technology. Since the mobile mental health application is similar to mobile applications nowadays, then it is easily adaptable by users in the current generation. Based on demographics, younger generations constitute most of the participants, which has led to the easy adaptation of the mobile application [60].

Consequently, SI was seen to be an important factor (55.2%) that has a positive significant influence on IU (β: 0.502; *p* = 0.020). It was indicated that the friends and family of a user find the application useful, that people around the users would be inclined in using the application, and available support for the advantage of using the mobile treatment from friends and family is available. This led to a significant and indirect effect on ASU (β: 0.137; *p* = 0.016) and PRU (β: 0.116; *p* = 0.016). Several studies verify this finding [53,59,62]. Their study explained how people around an individual influence their intention to use a certain technology, especially when in relation to health as people nowadays have already adopted the development of technology [63].

IU (54.4%) is another important factor that has significant positive influence on ASU (β: 0.596; *p* = 0.032). The indicators preceded that the availability of the mobile application would lead to people’s actual usage. Whenever users need remote medical care, it was indicated that they would be inclined to use the mobile application. Moreover, the applicability and intention of using the mobile application also lead to the actual use of the system. Thus, it was seen that a positive indirect effect of IU on PRU (β: 0.507; *p* = 0.016) was evident. Moreover, PU (50.7%) was seen to be one of the important and significant factors affecting IU (0.456; *p* = 0.010). People find it useful and advantageous when using the application in their lives, as there is a quick improvement and it provides useful services. This is supported by studies such as those by Tsai et al. [16] and Wu et al. [56], wherein there is acceptability for continuous patronage when the mobile application is beneficial to health-related activities.

Lastly, ASU was seen to be the least important factor (45.7%) but nonetheless a significant factor affecting PRU (0.850; *p* = 0.008). Based on the indicators, users would tend to use it daily and found it useful in coping with different situations such as with A negative mood, and that it acted as a motivator for daily activities. Following the studies of Gelderblom et al. [38] and Pee et al. [37], people perceive it to be of high usability when they continuously utilize a system or application. In addition, Wu et al. [56] explained how ASU is preceded by IU, leading to a positive PRU.

It was seen that the mobile mental health application helps users cope when they are overwhelmed by negative thoughts or in a depressive state and there is no one around to talk to or consult with. The ease of use, availability, and influence or support from others that users experience would lead to the actual use of the system and perceived usability of the mobile application. Moreover, its accessibility and helpfulness for coping, among its other advantages, have been evident upon using the application. Lastly, its constant availability and easy-to-use interface would promote inclination in its perceived usability and actual system usage. Thus, developers and stakeholders could consider the findings of this study to promote the availability and usage of mobile mental health applications to help others who are struggling with mental health issues. The benefits of remote consultation and check-ups were seen to be advantageous, not only in the Philippines, but could also be applied and extended worldwide.

### 5.1. Theoretical and Practical Contribution

Extending the technology acceptance model by considering factors such as social influence, service awareness, technology self-efficacy, convenience, user resistance, voluntariness, and perceived usability was seen to holistically measure the mobile mental health application available. The results present evident findings that would help developers and health practitioners promote the usage of health-related technologies such as mobile mental health applications. It was seen that when the accessibility and ease of use were positive, its perceived high usability would be evident among users.

Due to the increasing rate of people around the world suffering from mental health disorders or issues, aggravated by the COVID-19 pandemic, mobile mental health applications such as these would help mitigate anxiety, depression, even drug abuse, and death caused by the negative mental health. The adverse effects of mental health due to the lack of consultation, check-ups, and help lead to negative implications for a person’s mental, physical, and emotional state. The World Health Organization highlights the importance of taking care of one’s mental state to positively improve individual livelihoods. Thus, assessing applications such as this one would be beneficial for researchers, healthcare practitioners, and even users for promotion and mitigation worldwide.

### 5.2. Limitations and Future Research

Despite this study’s significant findings, several limitations were found. First, although this study was able to consider respondents from younger generations, due to the COVID-19 pandemic and a lack of resources, we were unable to obtain sufficient respondents to perform a more widespread data collection. Though online surveys would help predict outputs, a better spread of age groups could present other findings related to this study. Second, other factors were not considered aside from the ones mentioned. It is recommended that interviews be conducted to obtain qualitative results which may coincide with the quantitative results presented in this study. Moreover, other factors may be considered and applied based on the results of the interview. Third, user resistance may have been seen as less likely to be significant since an online questionnaire was administered. This indicates how the participants are inclined toward the use of technology. It is suggested that a similar study is conducted utilizing the traditional pen and paper survey, face-to-face interviews, and even group discussion. As such, possible reasons that were not discussed may be considered and analyzed. In addition, the inclusion of other factors may be considered to assess the usability of mental health applications. Fourth, it is suggested that methodology focusing solely on machine learning algorithm is considered in order to assess the claims found in several studies focusing on consumer behavior [64] and technology usability [65]. Lastly, differentiation based on demographics was not conducted. Clustering the results based on age group may promote findings to enhance the utility of the mobile mental health application available.

## 6. Conclusions

In recent years, mental health has emerged as one of the most important public health concerns. Mental illnesses such as depression, anxiety disorder, and mood disorder that have caused illnesses and even death by suicide have been prevalent among countries worldwide. In the Philippines, mental illness is the third most common disability. Despite the increasing number of mental health problems in the country, it is one of the problems that is given less attention. The COVID-19 pandemic has further aggravated this problem. Due to the increasing number of mental health issues, mobile mental health applications have emerged in the present time to help mitigate this unfortunate trend. This study aimed to assess the perceived usability of mobile mental health applications available in the Philippines.

Several factors such as social influence, service awareness, technology self-efficacy, perceived usefulness, perceived ease of use, convenience, voluntariness, user resistance, intention to use, actual use, and perceived usability were assessed in this study. Collecting data from a total of 251 respondents who were open to admitting that they have mental health issues and have utilized the mobile mental health application, an SEM–ANN hybrid was then conducted. The results showed that VO had the highest score of importance, followed by CO, PEOU, SA, SE, SI, IU, PU, and ASU.

It was seen that Filipinos are not fully aware of the existence of such mobile mental health applications and that these are effective in handling the mild to normal symptoms of mental health disorders. However, when presented with availability and accessibility, people would find it advantageous and beneficial for their mental health. People seemed to be open to adopting the technology, showed high levels of actual system use and perceived usability. This led to the insignificance of UR. This study contributes to the currently existing literature—especially that concerned with people with diagnosed and undiagnosed mental health disorders. This is highly beneficial in the current age since people are still adapting to the new environment shaped by the COVID-19 pandemic. Thus, developers and stakeholders could take advantage of this study for promotion and continuous patronage.

In addition, the result of this study can be used by government healthcare facilities to raise awareness of the existence of mobile mental health applications given that there is a scarcity of health facilities and workers in the country. Raising awareness among the Filipino population of such applications can be of help in dealing with individuals’ mental health. Finally, the results of this study could be applied and extended among other health-related mobile applications worldwide [63].

## Figures and Tables

**Table 1:** Demographic information of the participants.

Variable	Frequency	Percent (%)
Sex		
Female	128	51.00
Male	123	49.00
Age		
18 years old and younger	4	1.59
18–25	219	87.25
26–32	24	9.56
33–39	3	1.20
40–47	1	0.40
48 years old and older	0	0.00
Occupation		
Student	133	52.99
Working	118	47.01
Educational Attainment		
Primary	3	1.20
Secondary	57	22.71
Bachelor’s degree	173	68.92
Master’s degree	11	4.38
Doctorate degree	7	2.79
Access to Internet		
Yes	241	96.02
No	10	3.98
Use of Online Treatment in the past		
Yes	61	24.30
No	190	75.70

**Table 2:** Questionnaire.

Construct	Items	Measurement Items	References
Social Influence	SI1	Your friends and family think that mobile mental health applications are a useful thing.	Venkatesh et al. [39]
	SI2	Your friends and family think that mobile mental health applications would be useful for you.	Venkatesh et al. [39]
	SI3	Your friends and family would also use mobile mental health applications.	Venkatesh et al. [39]
	SI4	You often discuss the advantages of mobile treatment with your friends/family.	
	SI5	Your friends and family would be surprised if you use a mobile mental health application.	
	SI6	Your family and friends suggested that you use mobile mental health application.	Venkatesh et al. [39]
Service Awareness	SA1	You are aware of the existence of mobile mental health awareness.	Talukder et al. [18]
	SA2	You are aware of different available mobile metal health application.	Talukder et al. [18]
Self-efficacy	SE1	I feel confident finding information and advice in a mobile mental health application.	Meuter et al. [26]
	SE2	I have the necessary skills for using a mobile mental health application successfully.	Meuter et al. [26]
	SE3	I feel confident using the mobile mental health application regularly.	
Perceived Usefulness	PU1	I find mobile mental health to be useful to improve my life in general.	Venkatesh et al. [39]
	PU2	Using a mobile mental health application would improve my life quickly.	Venkatesh et al. [39]
	PU3	I would find mobile mental health applications useful.	Venkatesh et al. [39]
	PU4	I think that mobile mental health applications provide very useful services.	Venkatesh et al. [39]
Perceived Ease of Use	PEOU1	I find it easy to get the benefits from a mobile mental health application.	Venkatesh et al. [39]
	PEOU2	Using a mobile mental health application will be complicated.	Venkatesh et al. [39]
	PEOU3	Using a mobile mental health application will take a lot of effort.	Venkatesh et al. [39]
	PEOU4	I find mobile mental health applications are easy to use.	Venkatesh et al. [39]
	PEOU5	Learning to operate a mobile mental health application would be/is ease for me.	Venkatesh et al. [39]
	PEOU6	The interface of the mental health mobile application is user-friendly and fool-proof.	Venkatesh et al. [39]
Convenience	CO1	I find using the mobile mental health application convenient.	Shin [29]
	CO2	I can use the mobile mental health anywhere and anytime.	Shin [29]
	CO3	I can use the mobile mental health whenever needed in an undesirable situation.	Shin [29]
Voluntariness	VO1	I use mobile mental health application at my own will.	Tamilmani et al. [32]
	VO2	I was not forced by anyone to use the mobile mental health application.	Tamilmani et al. [32]
	VO3	I was introduced to use the mobile mental health application.	Tamilmani et al. [32]
User Resistance	UR1	I wouldn’t want the mobile mental health application to alter my traditional way of using health care services.	Tsai et al. [16]
	UR2	I wouldn’t want the mobile mental health application to interfere or change the way I interact with doctors.	Tsai et al. [16]
	UR3	Mobile mental health application can never replace the traditional therapy consultation.	Tsai et al. [16]
Intention to Use	IU1	Assuming that I was given the chance to access mental health mobile application, I intend to use its services.	Venkatesh et al. [39]
	IU2	Whenever I would need remote medical care from professionals, I would gladly use mobile mental health application services.	Venkatesh et al. [39]
	IU3	I intend to check the availability of a suited mobile mental health application.	Venkatesh et al. [39]
	IU4	I intend to use a mobile mental health application.	
Actual System Use	ASU1	I use mobile mental health applications daily.	Alam et al. [40]
	ASU2	I find mobile mental health applications useful when coping to different situation.	
	ASU3	Mobile health applications activities help lighten my mood and my state of mind.	Alam et al. [40]
	ASU4	Mobile health application motivates me in my daily life.	Alam et al. [40]
	ASU5	I encounter no problem when using the application.	
Perceived Usability	PRU1	Mobile mental health is useful during undesirable situations (e.g., racing negative thoughts, down mood).	Sonderegger and Sauer [41]
	PRU2	Mobile mental health applications help me cope.	Sonderegger and Sauer [41]
	PRU3	Mobile mental health applications are useful whenever there is no one I can talk to.	Sonderegger and Sauer [41]

**Table 3:** Indicators’ statistical analysis.

Variable	Item	Mean	StD	Factor Loading	
				Initial	Final
Social Influence	SI1	3.5538	0.82468	0.810	0.822
	SI2	3.5219	0.85470	0.848	0.876
	SI3	3.3825	0.92365	0.811	0.784
	SI4	2.7888	0.94193	0.548	0.507
	SI5	3.0996	1.03249	−0.276	-
	SI6	2.7769	0.91980	0.445	-
Service Awareness	SA1	3.6096	1.13796	0.929	0.915
	SA2	3.2550	1.18942	0.780	0.792
Technology Self-Efficacy	SE1	3.4980	0.86891	0.775	0.778
	SE2	3.6853	0.87667	0.772	0.770
	SE3	3.2829	0.84123	0.821	0.821
Perceived Usefulness	PU1	3.5976	0.78066	0.809	0.810
	PU2	3.2629	0.85941	0.665	0.665
	PU3	3.7610	0.76330	0.854	0.853
	PU4	3.8127	0.75947	0.856	0.856
Perceived Ease of Use	PEOU1	3.5458	0.71617	0.774	0.785
	PEOU2	2.6494	0.84652	−0.221	-
	PEOU3	2.6574	0.94770	−0.273	-
	PEOU4	3.5618	0.68642	0.791	0.785
	PEOU5	3.7012	0.74455	0.738	0.727
	PEOU6	3.4382	0.70368	0.751	0.762
Convenience	CO1	3.6693	0.71987	0.757	0.756
	CO2	3.7490	0.77250	0.891	0.891
	CO3	3.7251	0.80008	0.821	0.821
Voluntariness	VO1	3.7490	0.82750	0.765	0.766
	VO2	3.8645	0.81828	0.945	0.944
	VO3	1.9880	0.92296	0.712	0.713
User Resistance	UR1	3.1912	0.88277	0.858	-
	UR2	3.3984	0.87215	0.853	-
	UR3	3.5458	0.89045	0.539	-
Intention to Use	IU1	4.0040	0.71273	0.736	0.737
	IU2	3.9124	0.79516	0.742	0.742
	IU3	3.8406	0.78390	0.786	0.785
	IU4	3.6813	0.82098	0.801	0.800
Actual System Use	ASU1	2.4263	0.87495	0.518	0.550
	ASU2	3.4582	0.79577	0.739	0.742
	ASU3	3.3825	0.76755	0.818	0.807
	ASU4	3.1992	0.78496	0.724	0.723
	ASU5	3.2829	0.71251	0.426	-
Perceived Usability	PRU1	3.7331	0.80278	0.796	0.795
	PRU2	3.5339	0.75488	0.885	0.883
	PRU3	3.6972	0.78737	0.764	0.765

**Table 4:** Model fit.

Goodness-of-Fit Measures of SEM	Parameter Estimates	Minimum Cut-Off
Incremental Fit Index (IFI)	0.888	>0.80
Tucker–Lewis Index (TLI)	0.868	>0.80
Comparative Fit Index (CFI)	0.886	>0.80
Goodness-of-Fit Index (GFI)	0.876	>0.80
Adjusted Goodness-of-Fit Index (AGFI)	0.828	>0.80
Root Mean Square Error (RMSEA)	0.068	<0.07

**Table 5:** Direct, indirect, and total effects.

No	Variable	Direct Effect	*p*-Value	Indirect Effect	*p*-Value	Total Effect	*p*-Value
1	SE → PEOU	0.791	0.012	-	-	0.791	0.012
2	IU → ASU	0.596	0.032	-	-	0.596	0.032
3	SI → PU	0.502	0.020	-	-	0.502	0.020
4	PU → IU	0.456	0.010	-	-	0.456	0.010
5	SA → PU	0.341	0.003	-	-	0.341	0.003
6	CO → IU	0.300	0.009	-	-	0.300	0.009
7	PEOU → IU	0.266	0.036	-	-	0.266	0.036
8	VO → IU	0.596	0.011	-	-	0.596	0.011
9	ASU → PRU	0.850	0.008	-	-	0.850	0.008
10	SE → IU	-	-	0.210	0.026	0.210	0.026
11	SA → IU	-	-	0.155	0.003	0.155	0.003
12	SI → IU	-	-	0.229	0.009	0.229	0.009
13	CO → ASU	-	-	0.179	0.009	0.179	0.009
14	VO → ASU	-	-	0.152	0.012	−0.152	0.012
15	SE → ASU	-	-	0.125	0.030	0.125	0.030
16	SA → ASU	-	-	0.093	0.005	0.093	0.005
17	SI → ASU	-	-	0.137	0.016	0.137	0.016
18	PEOU → ASU	-	-	0.158	0.036	0.158	0.036
19	PU → ASU	-	-	0.272	0.018	0.272	0.018
20	CO → PRU	-	-	0.152	0.008	0.152	0.008
21	VO → PRU	-	-	0.129	0.010	−0.129	0.010
22	SE → PRU	-	-	0.106	0.028	0.106	0.028
23	SA → PRU	-	-	0.079	0.002	0.079	0.002
24	SI → PRU	-	-	0.116	0.016	0.116	0.016
25	PEOU → PRU	-	-	0.135	0.032	0.135	0.032
26	PU → PRU	-	-	0.231	0.012	0.231	0.012
27	IU → PRU	-	-	0.507	0.016	0.507	0.016

**Table 6:** Independent variable importance score ANN.

Factor	Normalized Importance
Social Influence	55.2%
Service Awareness	58.4%
Technology Self-Efficacy	56.0%
Perceived Usefulness	50.7%
Perceived Ease of Use	65.3%
Convenience	78.1%
Voluntariness	100%
Intention to Use	54.4%
Actual System Use	45.7%

## Data Availability

The data presented in this study are available on request from the corresponding author.
